# Epidemiology of bloodstream infections sustained by carbapenem-resistant Klebsiella pneumonia in a large teaching hospital in northern Italy

**DOI:** 10.1186/2047-2994-4-S1-P132

**Published:** 2015-06-16

**Authors:** C Alicino, DR Giacobbe, A Orsi, F Tassinari, C Trucchi, G Sarteschi, F Copello, V Del Bono, C Viscoli, G Icardi

**Affiliations:** 1 University of Genoa, Genoa, Italy; 2 IRCCS AOU San Martino - IST, San Martino, Italy

## Introduction

The rapid diffusion of carbapenem-resistant (C-R) *Klebsiella pneumoniae* (Kp) represents a global concern from both clinical and public health standpoints. In particular, bloodstream infections sustained by C-R Kp are associated with high mortality rates and the treatment of these clinical picture is a major challenge for clinicians.

## Objectives

To describe annual incidence of C-R Kp BSI, dating back to the first positive C-R Kp blood culture, in a 1,300-beds teaching hospital in northern Italy.

## Methods

We performed a retrospective study at IRCCS AOU San Martino – IST of Genoa, Italy. Between 1 January 2007 and 31 December 2014, overall hospitalizations and hospital patient-days were obtained from the hospital digital archives of patients’ clinical charts. Similarly, numbers of C-R Kp BSI were identified through the computerized microbiology laboratory database. Annual incidences of health-care associated C-R Kp BSI per 10,000 patient-days were calculated. Overall trends in the incidence of Kp BSI were recorded, by including annual incidences of carbapenem-susceptible (C-S) Kp BSI. Finally, 30-day survivals of both C-R and C-S Kp BSI were estimated.

## Results

From 2007 to 2014, we identified 511 episodes of Kp BSI, 349 of which caused by C-R Kp (68.3%). The overall incidence of C-R Kp BSI during the seven-year study period was 0.92/10,000 patient-days, with a peak of 1.77/10,000 patient-days in 2014. The highest annual incidences were registered in intensive care units, with a peak of 22.01 C-R Kp BSI/10,000 patient-days in 2012. The annual incidence of C-S Kp BSI remained steady throughout the study period. 30-day survival was significantly lower in C-R patients than C-S patients (Log-rank p = 0.002).

## Conclusion

In our hospital we observed a dramatic increase in the incidence of C-R Kp BSI occurred from 2009 to 2014. More efforts might be necessary to tackle the worrying C-R Kp diffusion in our hospital. Because of the dramatic shortage in antibiotics active against C-R Kp, further improvements in our infection-control practises are also of paramount importance to limit the high number of deaths due to C-R Kp BSI.

## Disclosure of interest

None declared.

